# Fabrication of Co_3_O_4_ from Cobalt/2,6-Napthalenedicarboxylic Acid Metal-Organic Framework as Electrode for Supercapacitor Application

**DOI:** 10.3390/ma14030573

**Published:** 2021-01-26

**Authors:** Ibnu Syafiq Imaduddin, Siti Rohana Majid, Shujahadeen B. Aziz, Iver Brevik, Siti Nor Farhana Yusuf, M. A. Brza, Salah R. Saeed, Mohd Fakhrul Zamani Abdul Kadir

**Affiliations:** 1 Centre for Ionics University Malaya, Department of Physics, University of Malaya, Kuala Lumpur 50603, Malaysia; ibnusyafiq@um.edu.my (I.S.I.); farhanayusuf@um.edu.my (S.N.F.Y.); 2 Advanced Polymeric Materials Research Lab., Department of Physics, College of Science, University of Sulaimani, Qlyasan Street, Sulaimani 46023, Iraq; mohamad.brza@gmail.com; 3 Department of Civil Engineering, College of Engineering, Komar University of Science and Technology, Sulaimani 46023, Iraq; 4 Department of Energy and Process Engineering, Norwegian University of Science and Technology, N-7491 Trondheim, Norway; 5 Charmo Research Center, Charmo University, Peshawa Street, Chamchamal, Sulaimani 46023, Iraq; salah.saeed@charmouniversity.org; 6 Centre for Foundation Studies in Science, University of Malaya, Kuala Lumpur 50603, Malaysia; mfzkadir@um.edu.my

**Keywords:** metal-organic frameworks (MOFs), TGA analysis, XRD and FESEM study, FTIR study, CV and GCD examination, electrochemical properties

## Abstract

In this study, cobalt-based metal-organic framework (MOF) powder was prepared via the solvothermal method using 2,6-naphthalenedicarboxylic acid (NDC) as the organic linker and N,N-dimethylformamide (DMF) as the solvent. The thermal decomposition of the pristine cobalt-based MOF sample (CN-R) was identified using a thermogravimetric examination (TGA). The morphology and structure of the MOFs were modified during the pyrolysis process at three different temperatures: 300, 400, and 500 °C, which labeled as CN-300, CN-400, and CN-500, respectively. The results were evidenced via field-emission scanning electron microscopy (FESEM), energy dispersive X-ray spectroscopy (EDX) and X-ray diffraction (XRD). The crystallite size of all samples was calculated using Scherrer’s equation. The smallest crystallite size of 7.77 nm was calculated for the CN-300 sample. Fourier transform infrared spectroscopy (FTIR) spectra were acquired for all the samples. The graphical study of the cyclic voltammogram (CV) gave the reduction and oxidation peaks. The charge transfer resistance and ionic conductivity were studied using electrical impedance spectroscopy (EIS). The galvanostatic charge–discharge (GCD) responses of all samples were analyzed. The relatively high specific capacitance of 229 F g^−1^ at 0.5 A g^−1^ was achieved in the sample CN-300, whereby 110% of capacitance was retained after 5000 cycles. These findings highlighted the durability of the electrode materials at high current densities over a long cycle.

## 1. Introduction

The increment of fossil energy consumption had pursued eco-friendlier and more sustainable related to high power energy sources to be created to replace the non-renewable resources [1,2]. At the same time, developing portable electronics, for example, mobile phones, tablets, cameras, power banks, and electric vehicles have encouraged the electrochemical energy storage device development such as batteries, fuel cells, and supercapacitors (SCs) to store and transport energy [3,4]. Previous reports established that porous carbon materials are crucial for energy storage application [1,2,3,4]. In the recent decade, porous carbon material was selected like the electrode material for electrochemical capacitors as a result of its large surface area of 3000 m^2^ g^−1^, chemical resistance, and high conductivity [5]. Nevertheless, the porous carbon capacity has arrived its bottleneck in getting the necessities of application in high energy storage [6]. Porous structure will provide extra space inside the electrode with a high surface area. Likewise, the additional spacious area stores more ion at the electrode/electrolyte interface, which is important for energy storage application.

To attain superior performance, carbon composites, which are well-defined as the addition of transition metal oxides or conducting polymers to carbon has become the replacement material to improve the cyclability and total capacitance over the contribution of the pseudocapacitive elements [1]. The fabrication of electrodes is imperative to enhance the electrochemical energy storage device performance [7,8]. Electrode materials have an essential role in the enhancement of high-performance SCs and batteries. Minakshi et al. [9] have used calcined chicken eggshell as an electrode material for application in battery and SCs. In another study, Minakshi et al. [7] have suggested that hybrid electrochemical energy storage devices made from biowaste material, such as eggshell, in combination with very inexpensive mixed metal oxides (NiO/Co_3_O_4_) are important and have a high potential for use in a wide variety of energy-intensive applications.

Metal-organic frameworks (MOFs) as a class of porous materials with crystalline in nature consist of metal ions that coordinated with organic ligands to produce one-, two-, or three-dimensional networks [10]. However, the need for improvements in conductivity, mechanical, and chemical stabilities have motivated researchers. This can be achieved via three approaches, namely: using pristine MOFs, where the electrolyte ions are physisorbed at the internal surface of the porous electrode material; forming nonporous carbon structures with a relatively high conductivity via MOF decomposition in an inert environment keeping a high specific surface area; heating the MOFs until the formation of metal oxides in air atmosphere. The electrochemical properties of MOFs have been determined as electrode materials for SCs, using transition metals, such as nickel and cobalt [11]. Diaz et al. [12] studied cobalt-based MOF (Co-MOF) with recording a specific capacitance of 0.47 F g^−1^ at 25 mV s^−1^. Moreover, MOF-derived nanoporous cobalt oxide gave 504 F g^−1^ at 5 mV s^−1^, which was carried out by Salunkhe et al. [13]. In another study, the pseudocapacitive (PSC) behavior of Co-MOF prepared in various solvents was examined by Ramachandran et al. [14]. The effect of solvents on the supercapacitive properties of Co-MOFs has also been shown to be important. The optimum specific capacitance was found to be 958.1 F g^−1^ when Co-trimesic acid MOF was used and prepared from N,N-dimethylformamide (DMF)/ethanol solvent.

The hybrid capacitors uniqueness is owing to the presence of pseudocapacitance behavior besides the electrical double-layer capacitance. These features offer quick and reversible Faradic reactions that cause large energy density and power density, which permits the hybrid capacitor to become a possible candidate for future electrical equipment [15]. The tuning electrochemical properties of MOF to achieve a higher specific capacitance than reported so far can be performed via proper heating to reach derivative MOF of cobalt oxide as a consequence of phase transformation. Herein, this work describes the electrochemical properties of MOF-derived cobalt(II,III) oxide (Co_3_O_4_) at different heating temperatures following the solvothermal process. The synergistic effect between the linker and Co_3_O_4_ metallic nature is expected to support the storage mechanism of the electrode.

## 2. Materials and Methods

### 2.1. Chemicals

The MOF was synthesized using the conventional solvothermal method according to reported works with modifications [16,17,18]. Of cobalt(II) nitrate 1.5 mmol (Nacalai Tesque, Kyoto, Japan) and 0.25 mmol of 2,6-naphthalenedicarboxylic acid (NDC) (6:1 metal:ligand mol ratio) (Sigma-Aldrich, St. Louis, MO, USA) were mixed in 30 mL of N,N-dimethylformamide (DMF) (Friendemann Schmidt, Parkwood, Australia) as the solvent. The mixture was stirred for 30 min until complete dissolution at room temperature, followed by heating in an VACUCELL oven at 150 °C (MMM group, Munich, Germany) for 12 h using a 100 mL teflon-lined stainless-steel autoclave (custom-made, Malaysia). Afterwards, the mixture was centrifuged at 5000 rpm for 5 min to make the product free from the solvent and then washed using ethanol (99.5%, Systerm Chemicals, Shah Alam, Malaysia) and dried to obtained Co-MOF powder (CN-R). 

### 2.2. Measurements

**Figure 1:**
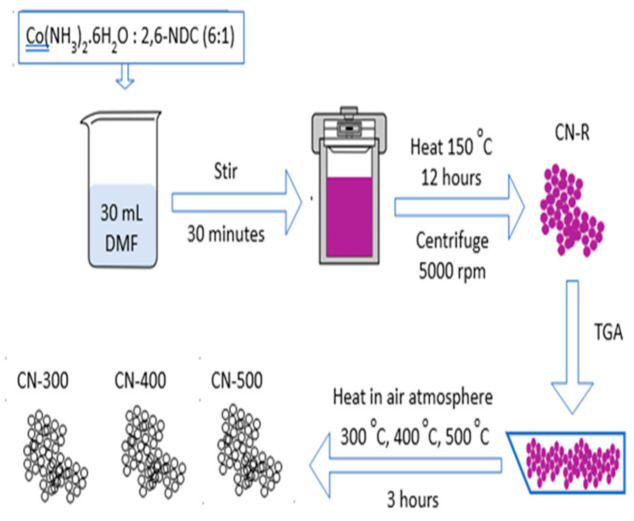
Procedure of the experiment.

The product was placed in an oven to evaporate the ethanol before implement analysis of cobalt-based metal-organic framework (MOF) (Co-MOF). The pyrolysis of Co-MOFs was carried out in air at three different temperatures for three hours to transform cobalt-based MOF into cobalt oxide derived from MOF as listed in Table 1. The temperatures were determined according to the thermogravimetric analysis (TGA) profile, which the sample was heated from 30 to 900 °C in N_2_ and air atmosphere at a rate of 10 °C min^−1^ by using the Mettler Toledo analyzer (TGA/SDTA851e as the main unit) (Mettler Toledo, Greifensee, Switzerland). The procedure of the experiment is shown in the flowchart (Figure 1). XRD was conducted using Panalytical Empyrean (Malvern Panalytical, Malvern, United Kingdom) with Cu K_α_ (λ = 1.54 Å) radiation with a step size of 40 kV and a time per step of 40 mA. FESEM images were captured and EDX was analyzed using Jeol JSM-7600S (JEOL, Tokyo, Japan) in high vacuum. Fourier transform infrared spectroscopy (FTIR) spectra were obtained using Nicolet iSIO smart FTIR (Thermo Fischer, Waltham, MA, USA) with the wavelength from 600 to 4000 cm^−1^. Viscous slurry consisting of active material, polyvinylidenedifluoride (PVdF) (Sigma-Aldrich, St. Louis, MO, USA), and carbon black powder, in N-methyl pyrrolidone (NMP) (Merck, Darmstadt, Germany) at an 8:1:1 mass ratio, was pasted on nickel foam serving as a current collector. Carbon black and PVDF were used to improve conductivity and bind the active material together, respectively [19]. Subsequently, in an oven, the coated 1 cm^2^ electrode was heated at 100 °C for 3 h before electrochemical measurements. The cobalt oxide derived from MOF was used as the working electrode and platinum (Pt) wire, and silver/silver chloride (Ag/AgCl) were used as counter and a reference electrode, respectively. In this experiment, the working electrode and counter electrode encompassed charging and discharging process. The reference electrode maintains the potential difference between itself and the working electrode during the timescale of the experiment within the supercapacitive cell. Cyclic voltammetry (CV), galvanostatic charge–discharge (GCD), and electrochemical impedance spectroscopy (EIS) were carried out and analyzed using Autolab potentiostat PGSTAT30 (Metrohm Autolab, Utrecht, The Netherlands). CV was conducted using 5, 10, 20, 30, 40, and 50 mV s^−1^ scan rate in −0.1–0.45 V potential window. GCD was tested using potential window between 0 and 0.45 V under 0.50, 0.75, 1.00, 1.25, and 1.50 A g^−1^ current density. For EIS, frequency from 10^4^ to 10^−2^ Hz was applied under the open-circuit voltage at 1 V potential amplitude. All the measurements were conducted in 3 M KOH as the electrolyte.

## 3. Results and Discussion

### 3.1. Thermal Analysis

**Figure 2:**
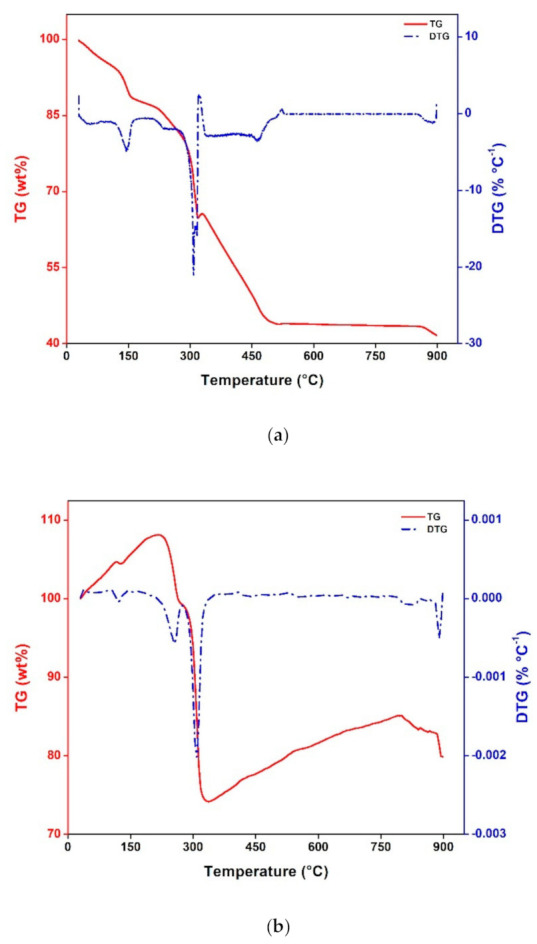
TGA of CN-R in (**a**) N_2_ and (**b**) air atmosphere.

A thermal decomposition study was conducted to analyze the designated heating temperatures for CN−R pyrolysis into metal oxide. From Figure 2a, three significant regions are highlighted. Firstly, a sharp decrease in sample weight can be observed in the 100 °C up to 150 °C region, which was caused by moisture vaporization due to the presence of water molecules (H_2_O), solvent (DMF), and alcohol in the sample. Secondly, an interesting thermal character is seen in which there is a significant weight loss from 300 °C until 319 °C, yielding a weight residue change from 76.71 to 64.77 wt % and further loss is seen until 500 °C (i.e., weight residue 44.16%). Based on the theoretical principle, in this region where cobalt hydroxide transforms into cobalt oxide [20]. Moreover, at 300 °C, NDC starts significant decomposing loss at a given temperature. Thirdly, there is a plateau with slight weight loss within 540–900 °C, and the weight loss is estimated to be about 2.23% of the original weight. This could be due to the sluggishness of the decomposition process of NDC and the termination of cobalt oxide transformation. Accordingly, the TGA pattern shows that the second region (300–500 °C) is the decisive region where one can obtain both the heating temperature of the decomposition of organic linkers and growth of cobalt oxide. From these two factors, it is easy to explain the value of specific capacitance of the CN-R sample.

The thermal decomposition of CN-R was also conducted in the air environment, as seen in Figure 2b. There is apparent mass gained for about 8% from 30 to 215 °C, which could be caused by several issues, such as the buoyancy effect, thermal expansion, electrostatic and magnetic forces, and atmospheric turbulence [21]. However, the dehydration process still can be noted from the DTG curve in the temperature range from 30 to 150 °C region, which is in the same region as TGA in N_2_ atmosphere. Another similarity of the thermal study in both environments can be seen in the range from 215 to 300 °C region. This region displays the decomposition evidence of 2,6-naphthalenedicarboxylate. A significant difference can be pointed out from this study is the weight loss recorded at 320 °C in which mass gain is observed from 330 to 800 °C (+10.87%). This observation mainly happened due to the contribution of oxygen in air that triggers oxidation reactions in the sample [22], which also confirmed the transformation of Co-NDC-MOF into cobalt oxide (Co_3_O_4_). Weight loss at a temperature higher than 800 °C was assigned to the termination of cobalt oxide transformation and the carbonization process occurred in the air environment. Lastly, much higher yield was achieved when the sample was heated in the air environment. Due to the similarities of cobalt transformation between 300 and 500 °C while there is apparent increasing mass in this region, and also the convenience of higher yield of cobalt oxide, heating temperature of 300, 400, and 500 °C in air was chosen to be tested for SCs application.

### 3.2. XRD Study

**Figure 3:**
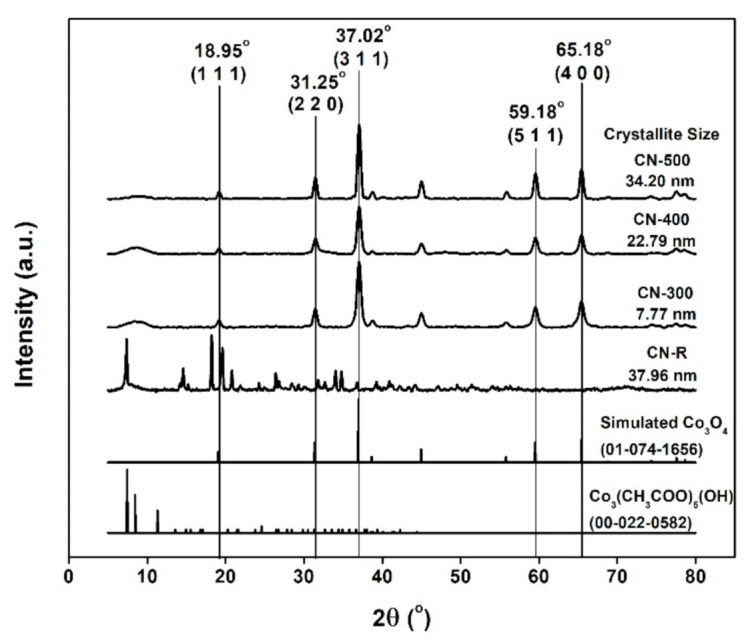
XRD spectra for CN−R, CN-300, CN-400, and CN-500.

In Figure 3, three distinct peaks of cobalt oxide samples after pyrolysis are observed at 2θ = 37.02°, 59.18°, and 65.18°, respectively. Importantly, these peaks are absent in the XRD pattern of the CN-R sample [23]. The obtained XRD patterns were compared with the simulated XRD patterns from the International Centre for Diffraction Data (ICDD) and showed a good agreement in the results. The most significant peak at 2θ = 37.02° was considered to measure the crystallite size of all the samples, using Scherrer’s Equation [24]:

(1) $$Τ=\;\frac{K\;\lambda }{\beta \cos\theta }$$

where β is the line broadening at full width half maximum (FWHM) intensity, *K* is a dimensionless shape factor, *λ* is the X-ray wavelength, *T* is the mean size of the crystalline domains, and *θ* is the Bragg’s angle. The smallest crystallite size of 7.77 nm was calculated for CN-300, thereby allowing the expectation that it could deliver a better performance and ease the ion transport [25].

### 3.3. FESEM Study

The field emission scanning electron microscopy (FESEM) as a robust technique for identifying the morphological appearance of nanostructured materials was acquired [26]. From the FESEM, both morphology and surface composition of metal frameworks were characterized using energy dispersive X-rays analysis (EDX) to an effort to support the XRD results.

**Figure 4:**
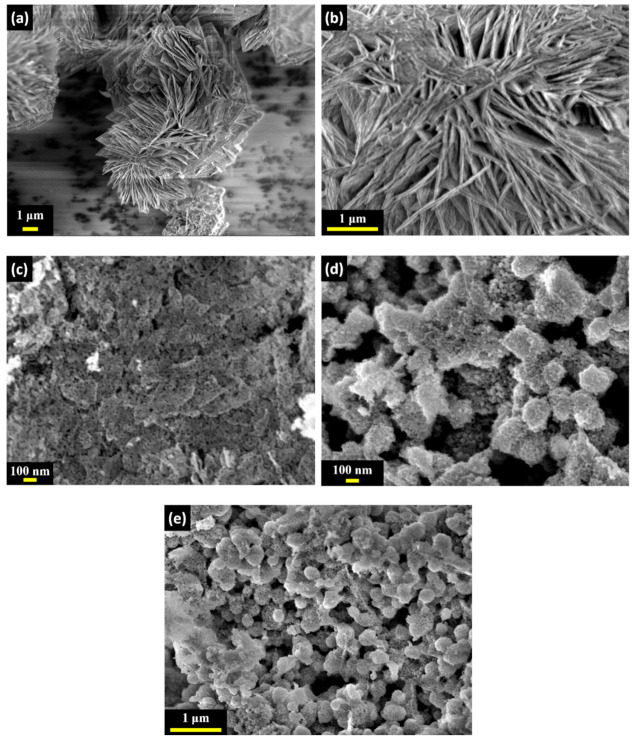
FESEM image for (**a**,**b**) CN-R, (**c**) CN-300, (**d**) CN-400, and (**e**) CN-500.

From Figure 4, the CN-R sample illustrates a plate-like structure and is transformed into a nanosize sphere when annealed at different temperatures as shown in Figure 4c–e. As the temperature increased the nanosize sphere in CN-400 and CN-500 showed a tendency to agglomerate. This could limit the accessible active surface area of the electrodes, leading to a lower specific capacitance in them.

**Figure 5:**
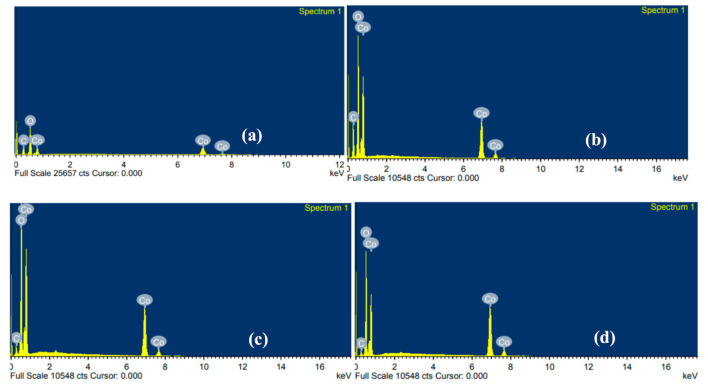
Composition of elements in (**a**) CN-R, (**b**) CN-300, (**c**) CN-400, and (**d**) CN-500.

Both Figure 5 and Table 2 show energy dispersive analysis X-ray analysis (EDX) spectra and the exact chemical composition, respectively. It is seen that the increment of temperature is effective on the composition of cobalt and carbon that behaved accordingly and disproportionately. Notably, it is also known that there is no existence of other elements as impurities in the samples. Interestingly, it is seen that a considerable quantity of cobalt exists. The noticeable intense peak reveals the presence of cobalt at high temperature.

### 3.4. FTIR Analysis

**Figure 6:**
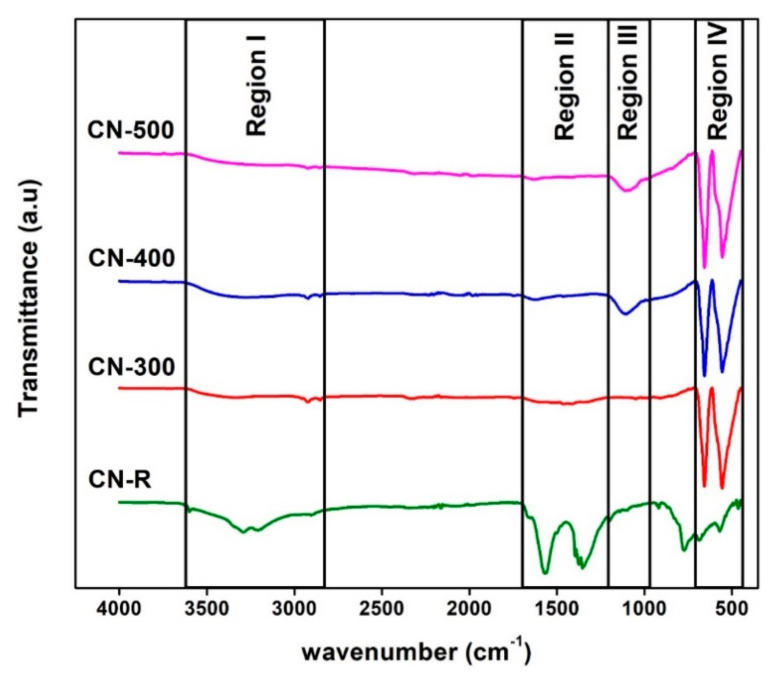
FTIR transmittance spectrum of all samples.

Figure 6 shows the FTIR outcomes and revealed the disappearance of C=O stretching and C=C stretching vibration bands at 1567 and 1355 cm^−1^, respectively, in the CN-R after being annealed at 300, 400, and 500 °C accordingly. Besides, at 3290 cm^−1^ [27], the hydrogen bonding band was vanished, which was due to the vaporization that occurred during heating and confirmed NDC decomposition. Similarly, a peak at 1095 cm^−1^ in CN-400 and CN-500 both appear due to C-H in-plane bend [28] and two sharp peaks centre at 657 and 553 cm^−1^, respectively. The metal-oxygen bonds are confirmed by the XRD, suggesting Co_3_O_4_ formation after pyrolysis. The appearance of the 657 cm^−1^ peak is evidence of existing vibration in ABO_3_; A and B represent the Co^2+^ and Co^3+^, respectively. On the other side, the peak at 553 cm^−1^ is ascribed to the vibration in OB_3_ vibration within the spinel lattice [29].

### 3.5. Electrochemical Properties

Cyclic voltammetry (CV) is an effective electrochemical method generally used to study the processes of oxidation and reduction of molecular species. In our previous works, the CV profile for the electric double-layer capacitors (EDLCs) device did not show the reduction and oxidation peaks [30,31,32,33,34]. Hegde et al. [35] fabricated carbon nanospheres (NSs) using a catalyst-free pyrolysis method from biowaste sago bark. The capacitive behavior of the carbon NSs verified by the CV curve at different scan rates, which showed nearly rectangular-like shape. The author indicated that the synthesized carbon NSs are suitable in the SCs electrode application.

**Figure 7:**
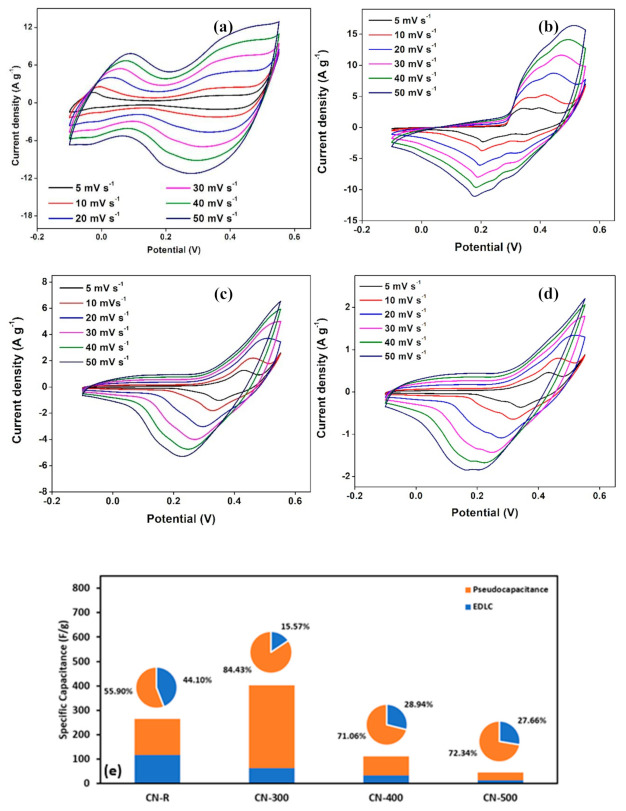
Cyclic voltammetry (CV) results of (**a**) CN-R, (**b**) CN-300, (**c**) CN-400, (**d**) CN-500, and (**e**) surface and diffusion contributions to the capacitance.

From Figure 7a–d, the voltammogram response contains two oxidation peaks at −0.02 V and 0.40 V and the reduction peaks at 0.13 V, and 0.35 V are recorded for CN-R at room temperature. After pyrolysis, CN-300 possesses oxidation peaks at 0.34 V and 0.40 V and reduction peaks at 0.21 V and 0.35 V. Differently, both CN-400 and CN-500 record only an oxidation peak at 0.43 V, and two reduction peaks at 0.35 V and 0.34 V, respectively. At high scan rates, both the oxidation and reduction peaks shifting to more positive and more negative sides, respectively. These can be due to a thin, diffuse layer and the internal resistance of the electrodes, namely the ohmic drop, *r_i_* [36]. The existence of redox peaks, even at high scan rates, also implies a great ability of the present material [14]. Presumably, the storage mechanism via pseudocapacitance (PSC) that can be realized from the CV curve of the samples, as explained below:

(i) For cobalt ions [37]:
${\mathrm{Co}}^{2+}{\;+\;3\;\mathrm{OH}}^{-}\;↔{\;\mathrm{CoOOH}\;+\;H}_{2}{O\;+\;e}^{-}$
${\mathrm{CoOOH}\;+\;H}_{2}O\;↔{\;\mathrm{Co}}^{4+}{\;+\;3\;\mathrm{OH}}^{-}{\;+\;e}^{-}$
(ii) For cobalt tetraoxide [38]:
${\mathrm{Co}}_{3}O_{4}{+H}_{2}{O+\mathrm{OH}}^{-}\;↔{\;3\;\mathrm{CoOOH}+e}^{-}$

**Figure 8:**
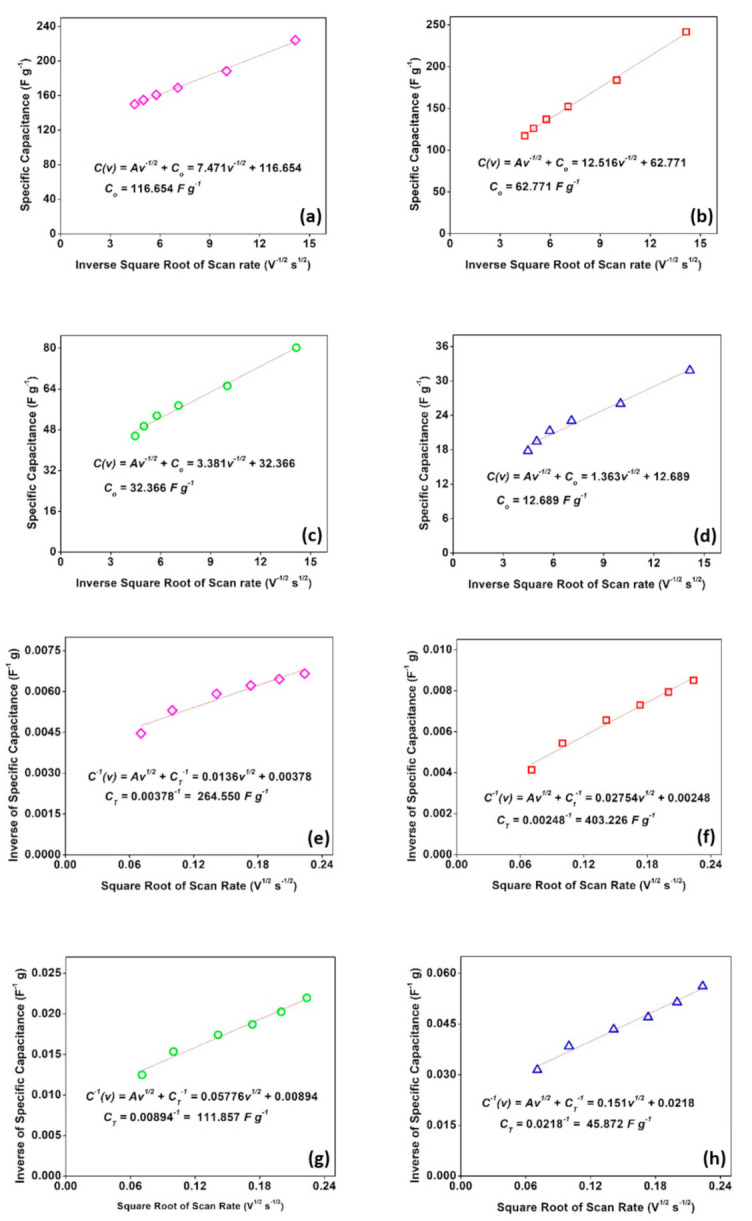
Trasatti method for C_o_ determination of (**a**) CN-R, (**b**) CN-300, (**c**) CN-400, and (**d**) CN-500; and C_T_ determination of (**e**) CN-R, (**f**) CN-300, (**g**) CN-400, and (**h**) CN-500.

Figure 7e displays a specific electrode capacitance for each sample, which is primarily assisted by the regulation of ions diffusion. Trasatti method was performed to compare both surface and diffusion contributions to the electrode’s specific capacitance. Total specific capacitance (C_T_) was calculated from Figure 8e–h. The y-intercept of the reciprocal of specific capacitance (C^−1^) against the square root of scan rate (V^1/2^ s^−1/2^) estimates the total specific capacitance (C_T_) of every sample in a three-electrode system. It was predicted that *C_T_* of CN-R, CN-300, CN-400, and CN-500 are 264.55, 403.23, 111.86, and 45.87 F g^−1^, respectively. Meanwhile, the y-intercept of specific capacitance (C) against the reciprocal of the square root of scan rate (V^−1/2^ s^1/2^) predicted the surface contribution to the specific capacitance (*C_o_*), which are 116.65, 62.77, 32.37, and 12.69 F g^−1^ for CN-R, CN-300, CN-400, and CN-500, respectively that illustrated in Figure 8a–d. Thus, the diffusion contribution to the specific capacitance, *C_i_*, can be calculated by:

(2) $$C_{i}=\;C_{T}-\;C_{o}$$

It is estimated that C_i_ for CN-R, CN-300, CN-400, and CN-500 were 147.90, 340.46, 79.49, and 33.18 F g^−1^, respectively. As mentioned above, all samples have a specific electrode capacitance that is predominantly regulated by ion diffusion, as shown in Figure 7e. Such control endorses the PSC and is the main reason for the high specific capacitance value obtained. Moreover, heated Co-MOFs were displaying much higher PSC behavior compared to the EDLC characteristics.

**Figure 9:**
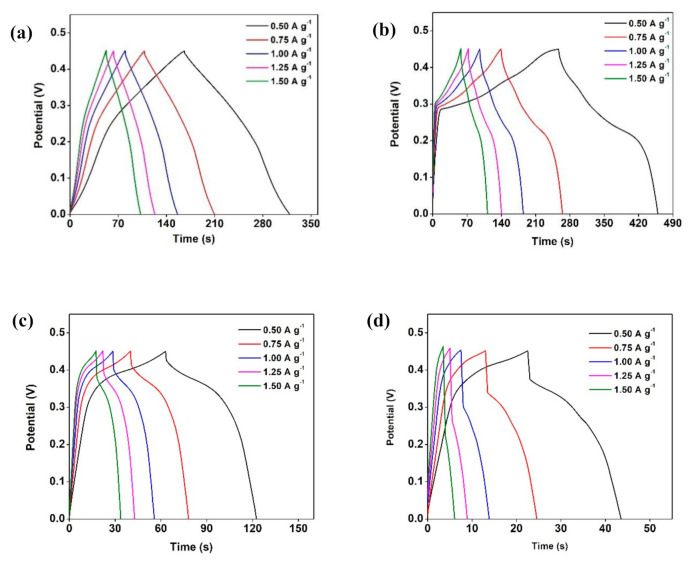

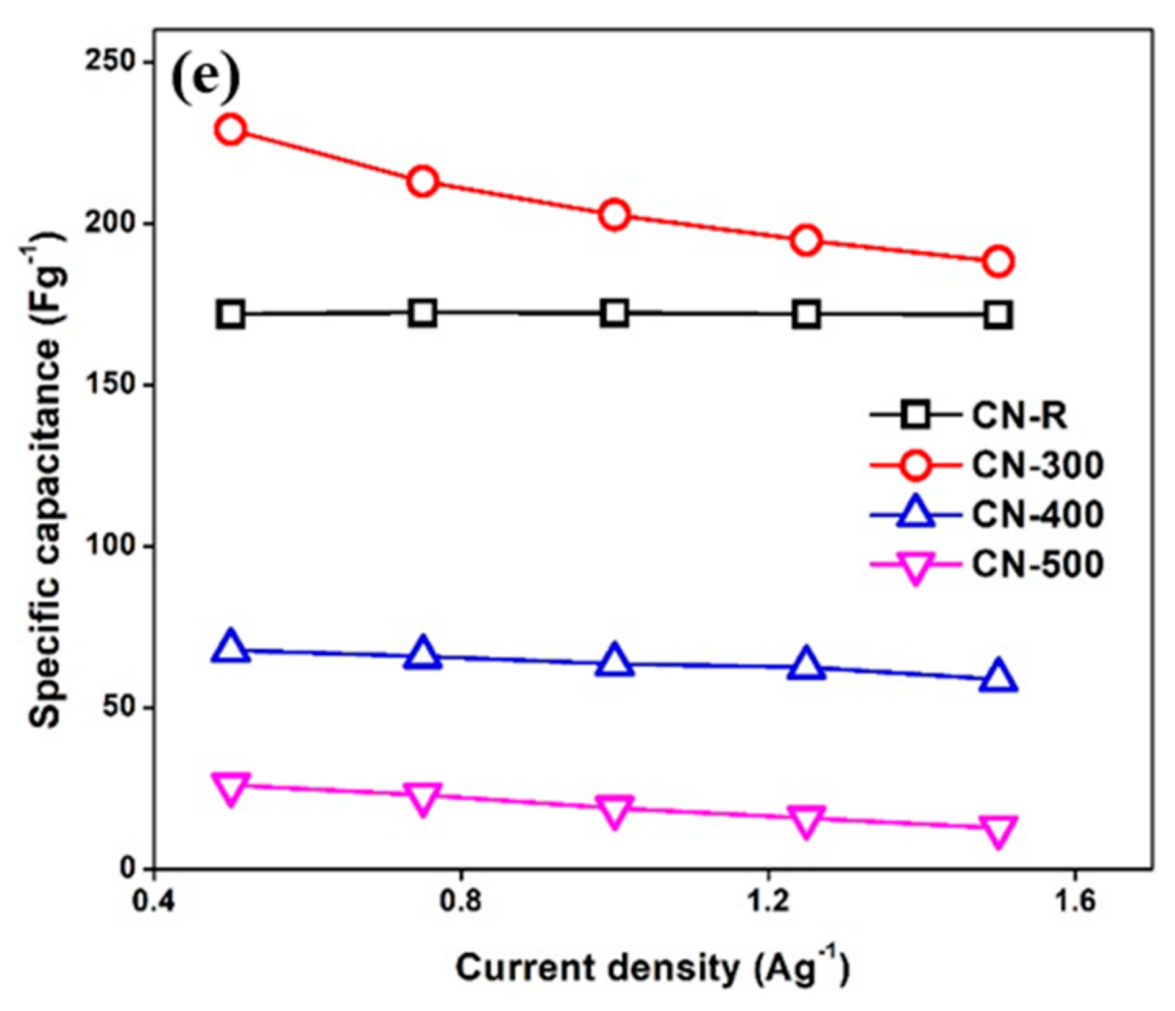
Galvanostatic charge–discharge (GCD) tests of (**a**) CN-R, (**b**) CN-300, (**c**) CN-400, (**d**) CN-500, and (**e**) specific capacitance at various current densities.

Moreover, Figure 9 illustrates the galvanostatic charge–discharge (GCD) curves of all samples. Large *r_i_* values were seen in CN-400 and CN-500 samples, which led to lower specific capacitance outcomes. This is due to the dense morphologies and agglomerations observed in the FESEM images. Besides, the CN-300 sample exhibited the longest discharge time and negligible *r_i_*, allowing it to achieve the highest specific capacitance of 229 F g^−1^ at the current density of 0.5 A g^−1^. Ali et al. [39] synthesized carbon NSs using lablab purpureus seeds, and they indicated that the carbon NSs materials are useful in SCs electrode applications. The authors determined that the lablab purpureus seeds fabricated at 800 °C indicated a specific capacitance of 300 F g^−1^ at the current density of 0.5 A g^−1^ and good stability even after 5200 cycles. The performance of the method used in this study is compared with other literature methods and are summarized in Table 3.

Niveditha et al. [40] prepared the Co_3_O_4_ electrode for supercapacitor (SC) application using a potentiodynamic (PD) approach. The Co_3_O_4_ electrode showed a good cyclic stability up to 1600 cycles and good charge retention. The Co_3_O_4_ electrode has a high specific capacitance of 396.67 F g^−1^ under the scan rate of 20 mV s^−1^. Tummala et al. [41] synthesized the Co_3_O_4_ electrode for SCs application using sol–gel and hydrothermal techniques. The Co_3_O_4_ electrode showed a specific capacitance of 162 F g^−1^ with a retention capacity of 72.2% after 1000 cycles for a specific current rate of 2.75 A g^−1^ in the 6 M KOH electrolyte. Jagadale et al. [42] prepared the Co_3_O_4_ electrode for supercapacitor application using a PD electrodeposition method. The Co_3_O_4_ electrode showed maximum specific capacitance of 365 F g^−1^ in 1 M KOH electrolyte at the scan rate of 5 mV s^−1^.

The CN-300 has shown not only relatively high specific capacitance but also high specific capacitance stability with 82.15% that stays constant between the lowest and highest current densities. In contrast, CN-R has a value of 99.90%.

In contrast, both CN-400 and CN-500 have relatively unsatisfactory specific capacitances. The specific capacitance values of the samples were in good agreement with the XRD crystallite size calculation in which the CN-400 and CN-500 had larger sizes than CN-300. Moreover, denser structure (agglomeration), larger crystallite sizes, and *r_i_* values could be associated with the relatively low specific capacitance values of CN-400 and CN-500.

### 3.6. Impedance Study

**Figure 10:**
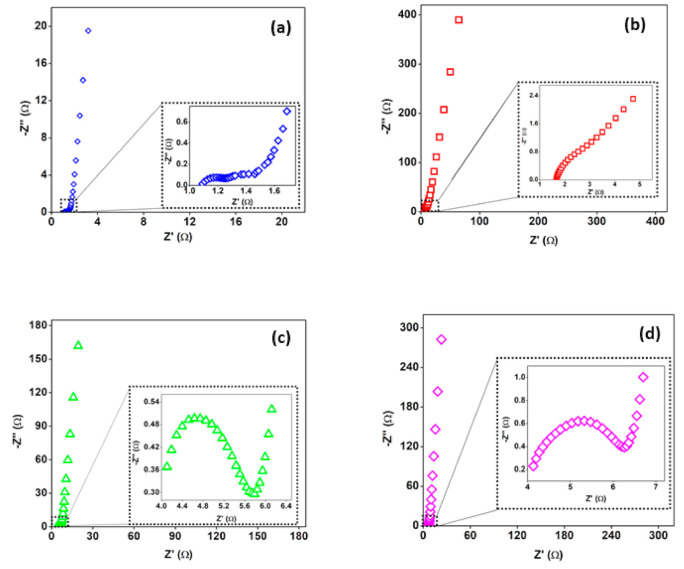
Electrical impedance spectroscopy (EIS) curves of (**a**) CN-R, (**b**) CN-300, (**c**) CN-400, and (**d**) CN-500.

Electrical impedance spectroscopy (EIS) is a non-destructive and a real technique, which is typically engaged in the study of electrolyte materials [45,46]. Aziz et al. [47] overviewed the EIS working principle. They demonstrated that the EIS is a method employed to investigate the electrical property of the bulk materials and their interfaces (electrode and electrolyte interfaces) within an extensive range of temperature and frequency. In EIS, the electrical resistance and ionic conductivity were studied. The ionic conductivity of the electrolyte can be obtained from the intersection of the real axis at the high-frequency region, which is the so-called series resistance (R_s_). From the analysis, the electron charge transfer resistance of the electrode (R_ct_) can be obtained from measuring the diameter of the semicircle response [48]. The Nyquist plot contains a semicircle at the high-frequency region that corresponds to charge-transfer resistance (R_ct_) and a straight line at the low-frequency region represents as presented in Figure 10. At the low-frequency range, the data point inclination must be 90°. However, due to the weakening of double-layer capacitance at blocking electrodes, there is an inclination in the EIS spectra. Another cause of this inclination is correlated with the ion transportation and surface disordering of the blocking electrodes [49,50,51].

The CN-R sample exhibited a lower R_ct_ than the CN-300, however, where O by the smaller NSs-like structures in CN-300 was believed to provide a larger accessible surface area. Ultimately, this enhanced its specific capacitance. Ali et al. [52] have synthesized porous nanocarbons (NCs) using biowaste oil palm leaves. The porous carbon nanoparticles (NPs) indicated higher super capacitance (SC) properties. In their study, low values of R_s_ and R_ct_ (0.39 Ω and 0.57 Ω, respectively) were obtained by fitting the impedance spectra. This indicates that these porous NCs are available as a precursor in the SCs electrode fabrication. In another study, Ali et al. [53] synthesized porous carbon NPs with a quite high SC value. Impedance spectra indicated low resistance values (R_s_ = 0.53 Ω, R_ct_ = 0.62 Ω) of these materials, representing their suitability for application in SC electrodes. Biswal et al. [54] employed modified ternary metal oxide (TO) as an electrode for SC applications. They showed that capacitance enhanced in the dodecyl trimethyl ammonium bromide (DTAB) existence as an additive. They employed Nyquist plot for the modified TO and showed that the incorporation of DTAB in the modified oxide not just reduced the R_s_ but also reduced the charge transfer resistance R_ct_ to the minimum value of approximately 0.5 Ω.

**Figure 11:**
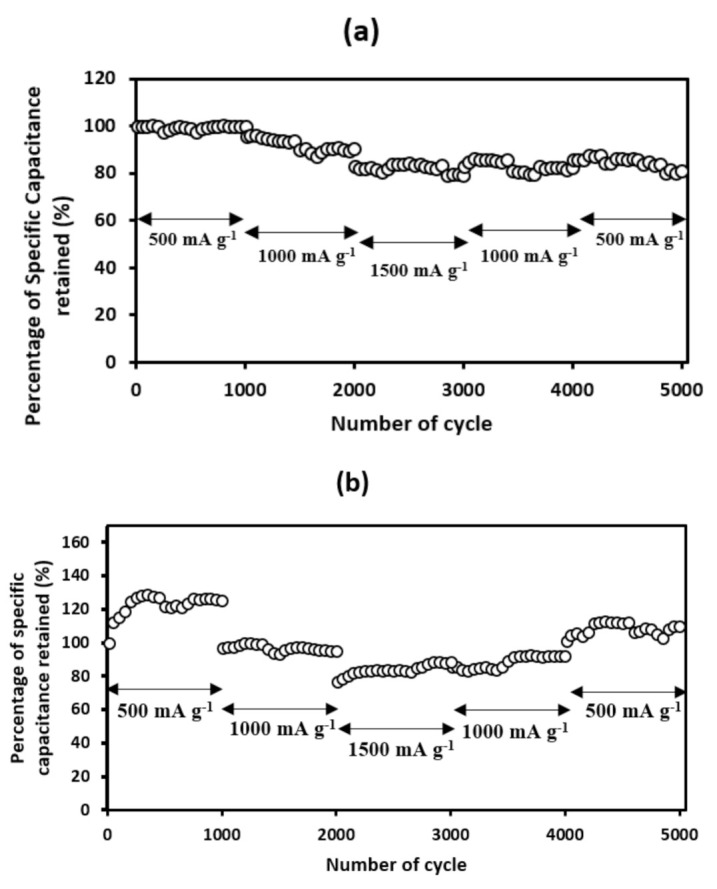
Rate capability plot for (**a**) CN-R and (**b**) CN-300.

Moreover, Figure 11a,b exhibits the rate performances of the CN-R and CN-300 at the current densities of 500, 1000, and 1500 mA g^−1^ for 5000 cycles accordingly. At 500 mA g^−1^, CN-R successfully retained 100% of its capacitance, while a 125% capacitance was retained in CN-300. When the current density was set to 1000 mA g^−1^, 90% and 77% of capacitance were recovered in the CN-R and CN-300 samples, respectively. Therefore, the excellent performance was achieved in CN-300 when the current density was readjusted to 500 mA g^−1^ after testing at 1500 mA g^−1^, thereby successfully recovering 110% of the specific capacitance retention after 5000 cycles. The ultrahigh rate capability of CN-300 could be observed due to the progressive activation of the surface, which opened more active sites for the electrolyte ions to penetrate the sample [55]. This allows a more extended cycle test for CN-300 to be possible.

## 4. Conclusions

An efficient approach of synthesis of Co-NDC-MOF and Co-MOF-derived cobalt oxide samples was carried out. The compatibility between the inorganic and organic components of the composite was confirmed by the coordination bond between them. The effects of temperature on the particle size during the pyrolysis of all samples were significant with the CN-300 sample showing the smallest crystalline size. The mechanism of charge storage within the supercapacitor system was realized via the preliminary electrochemical response. The electrochemical responses of the CN-300 were quite different from that of CN-400 and CN-500. The specific capacitance and surface morphology could be correlated. The XRD and surface morphology support each other during the data analysis. The increase of sharpness of crystalline peaks with a temperature rise was reflected from the small nanosize particles in FESEM appearance. The high specific capacitance of 229 F g^−1^ at 0.5 A g^−1^ was obtained in the CN-300 sample, whereby 110% of capacitance was retained after 5000 cycles. These results highlighted the electrode materials durability at high current densities over a long cycle.

## Figures and Tables

**Table 1:** Sample designation and applied temperature.

Temperature (°C)	Sample Name
No further heating	CN-R
300	CN-300
400	CN-400
500	CN-500

**Table 2:** Elemental composition of samples.

Sample	Elemental Composition (Atomic%)			Elemental Composition (Weight%)		
	Co	C	O	Co	C	O
CN-R	15.95	35.62	48.43	43.87	19.97	36.17
CN-300	18.27	36.37	45.36	48.08	19.51	32.41
CN-400	27.48	13.40	59.12	59.4	5.9	34.7
CN-500	37.81	10.62	51.57	70.05	4.01	25.94

**Table 3:** The performance of the method used in this study in comparison with other methods in the literature.

Metal	Organic Linkers	Methods	Solvents	Electrolyte	Current Density or Scan Rate	Highest Specific Capacitance (F g^−1^)	References
Cobalt zinc	Terephthalic acid	Solvothermal	DEF	0.1 M TBAPF_6_ in acetonitrile	25 m Vs^−1^	0.47	[12]
Cobalt	Terephthalic acid	Solvothermal (120 °C, 12 h)	DMF & ethanol,	3 M KOH	2 A g^−1^	958.1	[14]
			DMF & water			626.9	
			DMF, water & ethanol			428.3	
			DMF			393.4	
			Ethanol			654.4	
Cobalt	Terephthalic acid	Solvothermal (100 °C, 50 h)	DMF	1 M LiOH	0.6 A g^−1^	206.76	[43]
Cobalt	Hexamethylenetetraamine and 2,3,5,6- tetrafluoroterephathalic acid	Volatilization (RTP, several days)	Water	1 M KOH	1 A g^−1^	2474	[44]
					2 A g^−1^	1978	
Cobalt oxide	2-methylimidazole	Stirring, direct carbonation & oxidation	Methanol	KOH	5 mV s^−1^	504	[13]
Cobalt-MOF	2,6-naphthalenedicarboxylic acid	Solvothermal	DMF	3 M KOH	0.5 A g^−1^	229	This work

Where, DEF = N,N-diethylformamide, DMF = N,N-dimethylformamide, KOH = potassium hydroxide, LiOH = lithium hydroxide, TBAPF6 = tetrabutylammonium hexafluorophosphate.

## Data Availability

All data is contained within the article.
